# [Retracted] *In vivo* and *in vitro* effects of heme oxygenase-1 silencing on the survival of acute myelocytic leukemia-M2 cells

**DOI:** 10.3892/etm.2025.12909

**Published:** 2025-06-23

**Authors:** Sixi Wei, Yating Wang, Qixiang Chai, Qin Fang, Yaming Zhang, Jishi Wang

Exp Ther Med 9:931–940, 2015; DOI: 10.3892/etm.2015.2209

Following the publication of the above paper, it was drawn to the Editor’s attention by a concerned reader that, regarding the flow cytometric plots shown in Fig. 4A on p. 936, the data panels shown for the ‘BMNNC’ and ‘pRNAi-siHO-1’ experiments appeared to show similar groupings of dots, which would not have been anticipated if these experiments had been performed discretely under different experimental conditions, suggesting a fundamental flaw either in the way in which these experiments were performed, or in how the results were outputted.

The Editor of *Experimental and Therapeutic Medicine* has decided that this paper should be retracted from the Journal on account of a lack of confidence in the presented data. The authors were asked for an explanation to account for these concerns, but the Editorial Office did not receive a reply. The Editor apologizes to the readership for any inconvenience caused.

